# Mitochondrial genome provides species-specific targets for the rapid detection of early invasive populations of *Hylurgus ligniperda* in China

**DOI:** 10.1186/s12864-024-10011-z

**Published:** 2024-01-22

**Authors:** Chengjin Li, Buxin Wang, Yingchao Ji, Lan Huang, Xiaoyi Wang, Wenxia Zhao, Yanhong Wang, Hongyi Wang, Yanxia Yao

**Affiliations:** 1 Key Laboratory of Forest Protection of the National Forestry and Grassland Administration, Ecology and Nature Conservation Institute, Chinese Academy of Forestry, Beijing, 100091 China; 2 College of Plant Protection, Shandong Agricultural University, Tai’an, 271018 China; 3 Yantai Service Center of Forest Resources Monitoring and Protection, Yantai, 264003 China; 4 Shandong City Service Institute, Yantai, 264670 China

**Keywords:** *Hylurgus ligniperda*, Mitochondrial genome, Evolutionary rate, Molecular identification, Species-specific PCR

## Abstract

**Background:**

*Hylurgus ligniperda*, a major international forestry quarantine pest, was recently found to have invaded and posed a serious threat to the *Pinus* forests of the Jiaodong Peninsula in China. Continuous monitoring and vigilance of the early population is imperative, and rapid molecular detection technology is urgently needed. We focused on developing a single-gene-based species-specific PCR (SS-PCR) method.

**Results:**

We sequenced and assembled the mitochondrial genome of *H. ligniperda* to identify suitable target genes. We identified three closely related species for detecting the specificity of SS-PCR through phylogenetic analysis based on 13 protein-coding genes (PCGs). Subsequently, we analyzed the evolution of 13 PCGs and selected four mitochondrial genes to represent slow-evolving gene (*COI*) and faster-evolving genes (e.g. *ND2*, *ND4*, and *ND5*), respectively. We developed four species-specific primers targeting *COI*, *ND2*, *ND4*, and *ND5* to rapidly identify *H. ligniperd*a. The results showed that the four species-specific primers exhibited excellent specificity and sensitivity in the PCR assays, with consistent performance across a broader range of species. This method demonstrates the ability to identify beetles promptly, even during their larval stage. The entire detection process can be completed within 2–3 h.

**Conclusions:**

This method is suitable for large-scale species detection in laboratory settings. Moreover, the selection of target genes in the SS-PCR method is not affected by the evolutionary rate. SS-PCR can be widely implemented at port and forestry workstations, significantly enhancing early management strategies and quarantine measures against *H. ligniperda*. This approach will help prevent the spread of the pest and effectively preserve the resources of Chinese pine forests.

**Supplementary Information:**

The online version contains supplementary material available at 10.1186/s12864-024-10011-z.

## Background

The biological invasion of forests poses a significant challenge to biodiversity and forestry resources and constitutes a widespread global phenomenon [1, 2]. Major invasive species have substantially damaged Chinese forestry resources, adversely impacting species diversity and ecological stability and surpassing native species [3, 4]. Consequently, studying potentially invasive species has become crucial in contemporary forestry research [5]. Rapid detection and identification of invasive species are indispensable and form the foundation for developing effective management strategies and quarantine measures [6, 7].

Bark beetles represent a highly diverse group of insects and are counted among the most destructive pests in forest ecosystems, particularly when introduced beyond their native habitats [8, 9]. Pines are favored hosts for several bark beetle species known for their destructive potential [10]. The red-haired pine bark beetle, scientifically known as *Hylurgus ligniperda* (Coleoptera: Curculionidae), is a well-documented forest insect that primarily infests pine species [11]. The *H. ligniperda* beetle typically targets the inner bark of subhealthy pine trees, often near the base of the trunk or roots, and threatens live trees and seedlings in densely populated areas [12, 13]. Additionally, *H. ligniperda* is a vector for plant pathogenic fungi and wood-degrading fungi, leading to trunk disease [12, 14, 15]. This situation has had significant implications for the import and export of pinewood, prompting some countries to implement stringent quarantine measures employing various methods to curb the spread and introduction of *H. ligniperda* [16, 17].

Owing to its small size, the *H. ligniperda* beetle often remains concealed beneath the bark of pinewood [18]. This concealment enabled *H. ligniperda* to mate with its siblings before dispersal, thereby increasing the likelihood of successful colonization [19]. Like many other beetles, morphological differentiation remains even more implausible for its immature stages. China is a major consumer of timber and imports wood from diverse sources, some of which have suffered severe damage from *H. ligniperda* infestations [18, 20]. Since July 2019, *H. ligniperda* has been sporadically detected in traps in Tai’an, Weihai, and Yantai cities in Shandong Province, China [21]. It was not until October 2020 that researchers identified a significant population of *H. ligniperda* in the roots of *Pinus thunbergii* within the coastal shelter forests of Muping District, Yantai city, confirming the invasive presence of *H. ligniperda* in China [11]. The invasion of *H. ligniperda* has led to the degradation of the windbreak and sand-fixing abilities of coastal shelter forests, posing a significant threat to Chinese pine forests due to the strong dispersal capabilities of *H. ligniperda*.

**Fig. 1:**
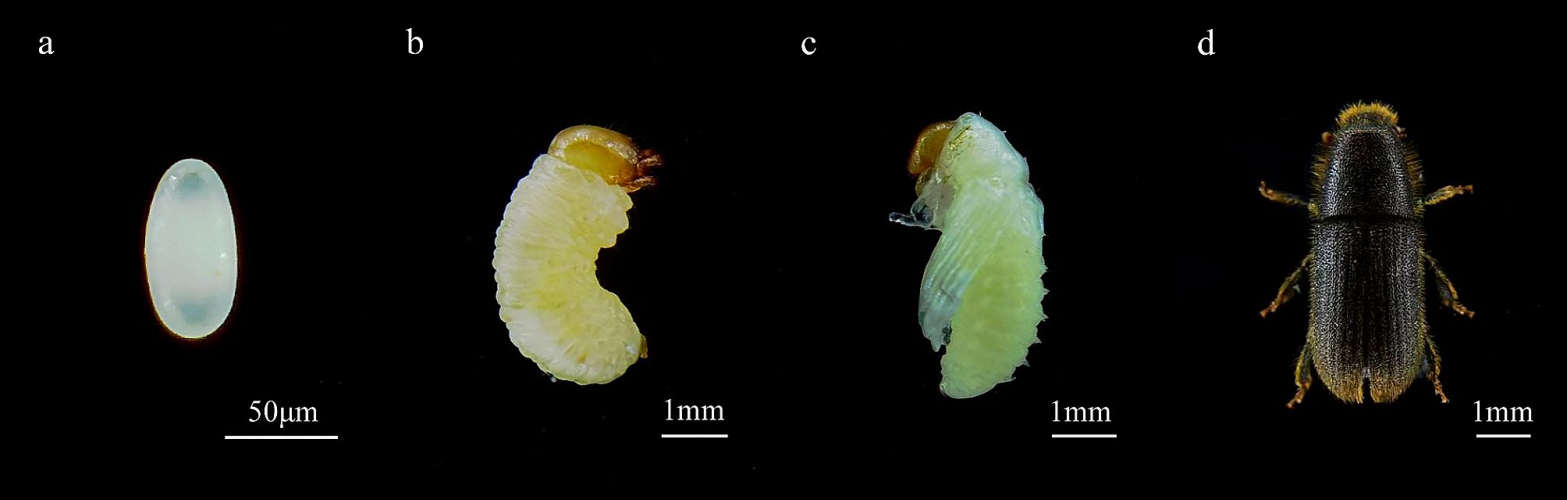
Morphological characteristics of *Hylurgus ligniperda* at four developmental stages. **a**: Egg; **b**: Larva; **c**: Pupa; **d**: Adult

In China, records of *H. ligniperda* have been relatively scarce, mainly consisting of reports related to port interceptions. Grassroot personnel often lack the necessary experience for accurate identification and typically require the assistance of insect taxonomists to confirm the presence of *H. ligniperda*. Identifying the egg, larval, and pupal stages is particularly challenging, often necessitating the rearing of specimens until they reach the adult stage for morphological identification (Fig. 1) [22]. Stage-specific taxonomic keys, phenotypic variability in key characteristics, and damaged adult specimens further complicate the identification process [23, 24]. Although morphological identification complemented by DNA barcoding can be employed, this method demands taxonomic expertise, is time consuming, and is constrained by the scope and accuracy of the existing DNA database [25]. In contrast, single-gene-based species-specific PCR (SS-PCR) is an efficient and reliable molecular method widely used to detect economically significant and invasive insects [4, 24, 26–29].

Mitochondrial genes are prederred targets for the development of SS-PCR, as mitochondrial genes are commonly regarded as maternally inherited genes that maintain compositional conservation and stable acquisition [30, 31]. Advances in sequencing technology have led to the rapid expansion of mitochondrial genome databases. This proliferation has opened up new possibilities for identifying targets suitable for species-specific primers. Several mitochondrial genome studies have demonstrated that regions exhibiting substantial nucleotide variations are often valuable as specific markers for interspecific species identification. These markers are often characterized by a high evolutionary rate [32, 33]. However, almost all research on SS-PCR has focused on the *COI* gene as a target, which is typically used as a universal DNA barcode for species classification, identification, and phylogenetic relationship assessment, even though its evolutionary rate is often the slowest. The relationship between target genes and evolutionary rates remain unclear, and we hope our study will provide some insights into this matter.

In the present study, we focused on the challenges of being unable to identify significant threats of *H. ligniperda* to forestry of China quickly and accurately. The mitochondrial genome provides SS-PCR target genes for rapid identification and monitoring of early invasive populations of *H. ligniperda* while also attempting to investigate the correlation between target genes and gene evolutionary rates. Moreover, it supports quarantine and prevents pest proliferation as a generalized molecular technique.

## Results

### **General features of the** ***Hylurgus ligniperda*** **mitochondrial genome**

**Fig. 2:**
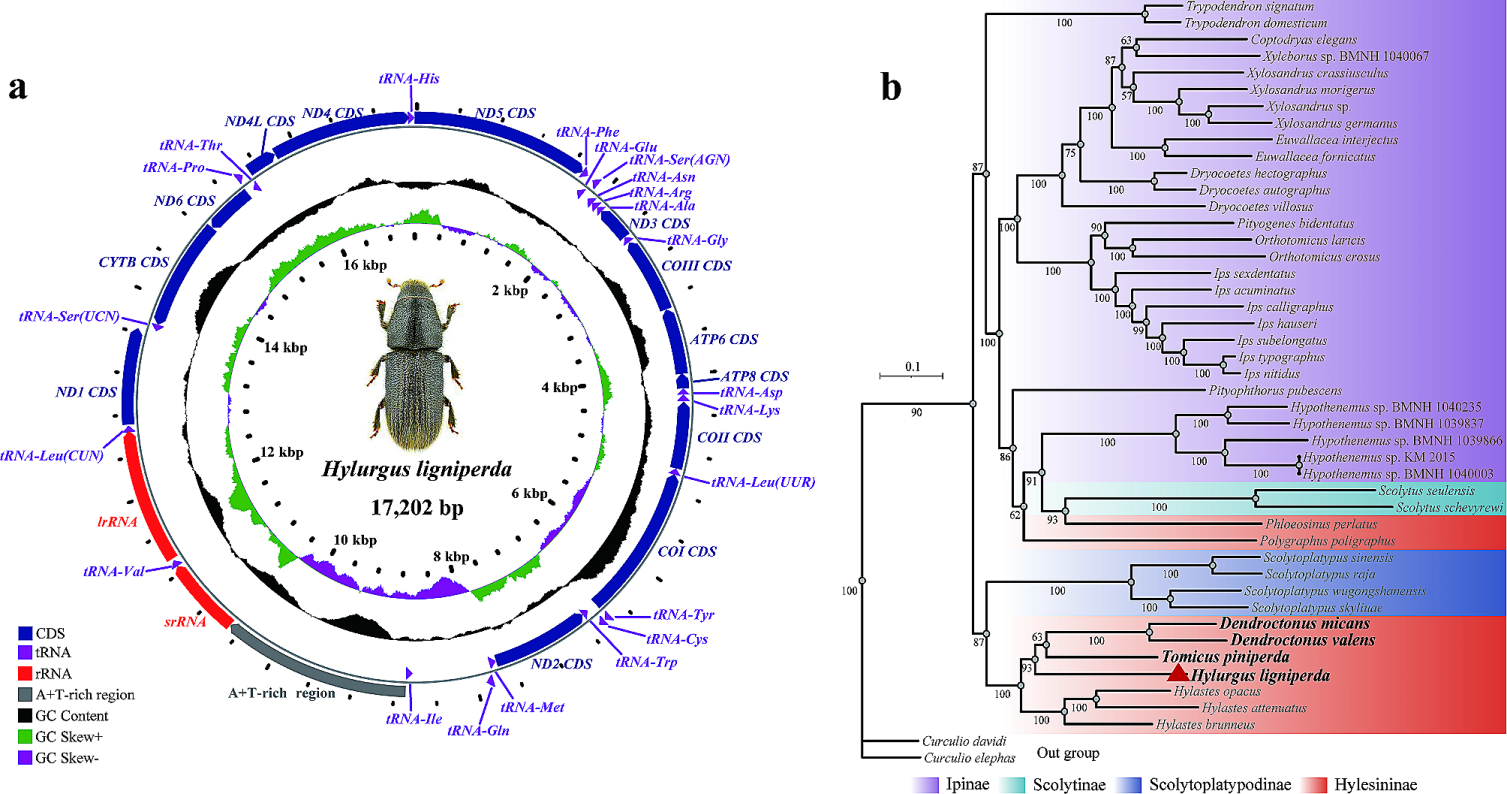
Genetic map of the complete mitochondrial genome of *Hylurgus ligniperda***(a)** and the phylogenetic tree of *Hylurgus ligniperda* inferred from 44SPE using maximum likelihood **(b)**

Two identical complete mitochondrial genomes for each sample were obtained using Geneious Prime 2020 and IDBA-UD assembler software. The different assembly methods produced the same structures. The results for the Weihai (WH) and Yantai (YT) samples were consistent by comparing the mitochondrial genome sequence and gene positions of specimens from four sites. The 36 gene sequences of the mitochondrial genomes from the four sites were identical. In contrast, the 16 S gene of the Qingdao (QD) sample was 2 bp longer than that of the other sample, and the *tRNA*^*Ile*^ positions of the Tai’an (TA) and QD samples differed from those of the other two samples. Since two of the four samples shared the same mitochondrial genome sequence with a length of 17,202 bp (Fig. 2a), they were used for analysis and were assigned the GenBank accession number (GenBank: OR105874).

The whole mitochondrial genome of *H. ligniperda* is circular and double-stranded, including 37 coding genes (13 PCGs, two rRNA genes, and 22 tRNA genes). A putative control region, in which there were nine PCGs and 14 tRNA genes, was encoded by the majority strand (J-strand). The remaining genes were encoded by the minority strand (N-strand) (Table S2). The mitochondrial genome of *H. ligniperda* included a total of 97 bp of overlapping bases between 16 adjacent genes, with a maximum overlap of 29 bp located at *COI*-*tRNA*^*Try*^ and a minimum of 1 bp located at seven junctions (*tRNA*^*Arg*^-*tRNA*^*Ala*^, *tRNA*^*Ala*^-*ND3*, *COIII*-*ATP6*, *ATP6*-*ATP8*, *tRNA*^*Asp*^-*tRNA*^*Lys*^, *tRNA*^*Cys*^-*tRNA*^*Trp*^, *tRNA*^*Ser*(*UCN*)^-*CYTB*, and *CYTB*-*ND6*). The total apparent length of the intergenic spacers was 811 bp, and the spacers were dispersed between 13 adjacent genes. The smallest gene spacer (2 bp) was located at *ND6*-*tRNA*^*Pro*^, whereas the longest (715 bp) was inserted at *tRNA*^*Gln*^-*tRNA*^*Ile*^. The *tRNA*^*Ile*^ divides the spacer region of *12 S* between *tRNE*^*Gln*^ into two parts of different lengths (both are often long), similar to most other bark beetle species. The base components were A (36.3%), T (40.6%), G (14.4%), and C (8.8%), with an A + T content of 76.9%, indicating significant AT bias. The nucleotide skew analysis revealed a negative AT-skew (− 0.0560) and a positive GC-skew (0.2414) (Table S3).

The total length of the 13 PCGs was 11,164 bp within the mitochondrial genome. Among these genes, *ATP8* was the shortest gene (156 bp), and *ND5* was the longest (1,714 bp). The 13 PCGs initiate with the ATN start codon (six ATTs, five ATGs, and two ATAs). Eleven PCGs stopped with TAA and TAG (only *ND4L*) and *ND5* truncated termination codons with a single T (Table S2). All 22 tRNA genes are discontinuously interspersed between the rRNA genes and PCGs in the mitochondrial genome, with lengths ranging from 63 bp (*tRNA*^*Ala*^ and *tRNA*^*Asp*^) to 69 bp (*tRNA*^*Gln*^). The control region in the mitochondrial genome of *H. ligniperda* located between *tRNA*^*Ile*^ and *srRNA* is the largest noncoding region, with a length of 1,821 bp.

### Phylogenetic analyses

The analysis of 13 PCGs within 44SPE, using IQ-TREE and MrBayes, produced two trees with nearly identical topologies featuring a few unstable branches (Fig. 2b and Fig. S1). Our primary focus was elucidating the relationships between *H. ligniperda* and its closely related species. The phylogenetic results unequivocally support the clade ((*H. ligniperda* + (*T. piniperda +* (*D. micans* + *D. valens*))) + (*Hylastes brunneus +* (*Hylastes opacus* + *Hylastes attenuatus*)) with high support values in both ML and BI analyses (Fig. 2b and Fig. S1). Remarkably, traditional taxonomic categorization places *Phloeosinus perlatus* and *Polygraphus poligraphus* in the Hylesininae subfamily. However, our molecular analysis consistently clusters them with Scolytinae and Ipinae, which aligns with previous findings [34–37].

We also conducted genetic distance analyses based on the 13 PCGs among the 9SPE, which supported our phylogenetic relationships (Fig. S2). Notably, the smallest genetic distance was observed between *D. valens* and *D. micans* (0.183), while the greatest distance was between *P. perlatus* and *D. valens* (0.414), or between *P. perlatus* and *Hylastes attenuatus* (0.414). Six species exhibited close genetic proximity to *H. ligniperda*, except for *P. perlatus* and *Polygraphus poligraphus*.

Both phylogenetic relationships and genetic distance analyses highlight the diagnostic value of molecular data within Hylesininae, even in the presence of morphological similarities. The 4SPE within this group appear to be more closely related. This result indicated they could be an initial control species for primer-specific tests.

### Evolutionary rates and selection of SS-PCR target genes

**Fig. 3:**
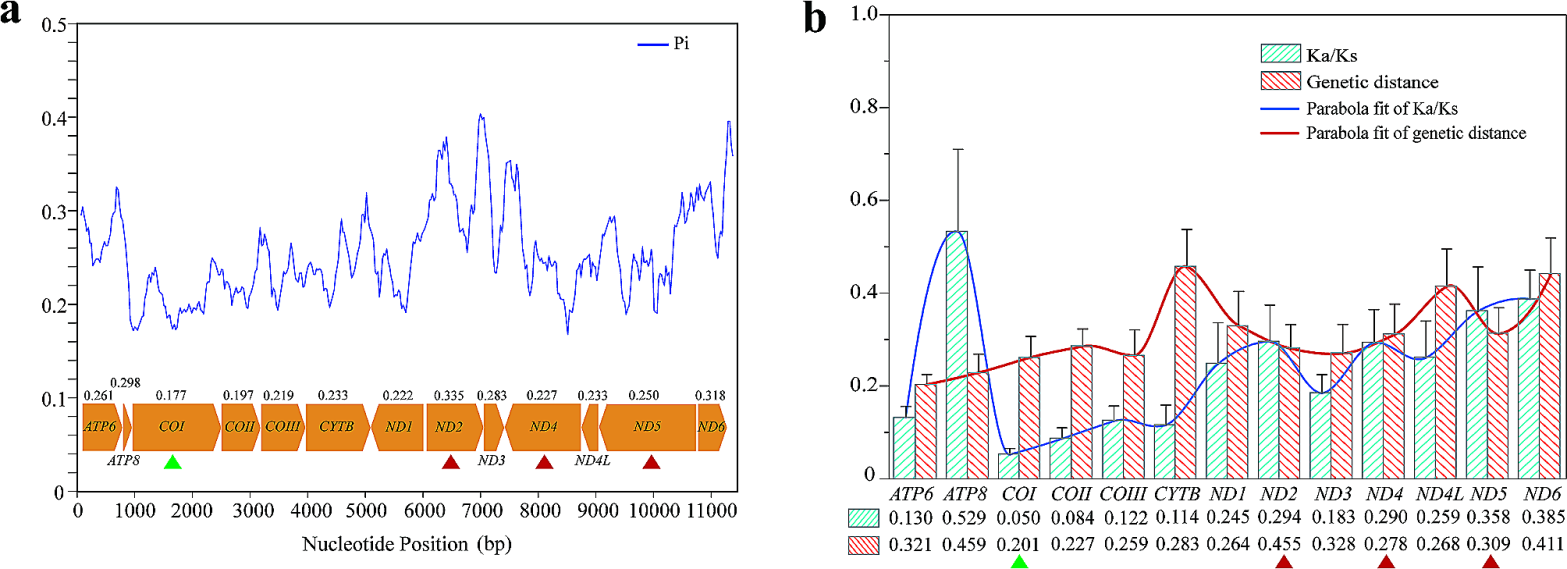
Gene evolutionary rate analysis. **(a)** Sliding window analysis revealing nucleotide diversity. **(b)** Mean genetic distances and Ka/Ks of mitochondrial gene sequences. The triangles represent the selection of target genes. The red and green colors represent the fast and slow evolutionary rates, respectively

In order to identify more effective targets for the molecular detection of *H. ligniperda*, we assessed the evolutionary patterns of PCGs in the 4SPE. Our analysis considered parameters such as Pi, Ka/Ks, and genetic distance for each PCG (Fig. 3a and b). The average Pi of individual genes ranged from 0.177 (*COI*) to 0.335 (*ND2*). Notably, the three high-ranking genes with the highest Pi values were *ND2* (0.335), *ND6* (0.318), and *ATP8* (0.298), while those with lower Pi values included *COI* (0.177), *COII* (0.197), and *COIII* (0.219). The average Ka/Ks ratio was a diagnostic indicator for detecting molecular adaptation and was utilized to assess the evolutionary rate of each PCG in the 4SPE. The average Ka/Ks values varied from 0.050 (*COI*) to 0.529 (*ATP8*) and consistently remained below one, indicating that all PCGs have undergone purifying selection. Specifically, these genes had high evolutionary rates, such as *ATP8* (0.529), *ND6* (0.385), and *ND5* (0.358), or low evolutionary rates, such as *COI* (0.050), *COII* (0.084), and *CYTB* (0.114). The average genetic distance among PCGs followed a similar pattern, with genes exhibiting fast evolutionary rates, including *ATP8* (0.459), *ND2* (0.455), and *ND6* (0.411), and genes with slower evolutionary rates, such as *COI* (0.201), *COII* (0.227), and *COIII* (0.259).

Overall, the *COI* gene is widely recognized as a suitable target for interspecific taxonomy, exhibiting the lowest evolutionary rate and resulting in the smallest nucleotide differences among closely related species. In our attempt to explore the relationship between evolutionary rates and the selection of target genes for primer design, we aimed to choose at least three genes with relatively rapid evolutionary rates. Long nucleotide sequences might also offer a wealth of target regions, benefiting from our primer design (Fig. 3a and b). Finally, apart from the *COI* gene, we also selected *ND2*, *ND4*, and *ND5* to represent genes with relatively rapid evolutionary rates.

### Specificity, sensitivity, and stability test of SS-PCR method

**Fig. 4:**
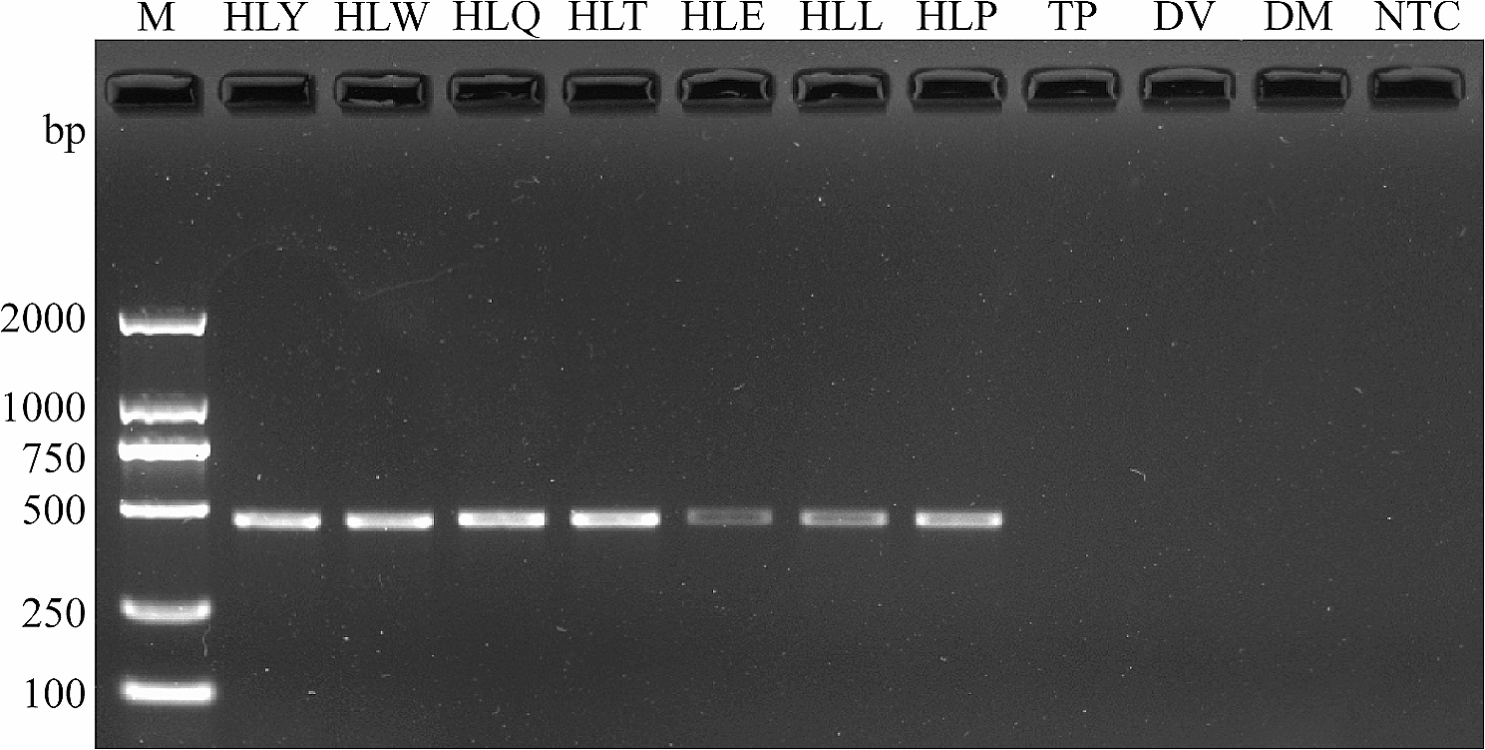
Specificity and stability of the primers targeting the *COI* gene. M: DL2000 DNA marker; HLY: *H. ligniperda*, Yantai; HLW: *H. ligniperda*, Weihai; HLQ: *H. ligniperda*, Qingdao; HLT: *H. ligniperda*, Tai’an; HLE: *H. ligniperda*, egg; HLL: *H. ligniperda*, larvae; HLP: *H. ligniperda*, pupae; TP: *T. piniperda*; DV: *D. valens*; DM: *D. micans*; NTC: no template control

We initially used universal primers to amplify the DNA barcoding of four species and ensured the accuracy and suitability of all samples (Fig. S3). We targeted the *COI*, *ND2*, *ND4*, and *ND5* genes and screened primers with excellent performance. We conducted specific detection experiments among the 4SPE using only four primers in the *H. ligniperda* sample case. All four primers successfully amplified fragments of lengths of 474 bp (*COI*), 434 bp (*ND2*), 346 bp (*ND4*), and 452 bp (*ND5*), respectively (Fig. 4 and Fig. S4). Furthermore, these four primers demonstrated stability in their performance when applied to *H. ligniperda* from various geographical populations (YT, WH, QD, and TA) and different developmental stages (egg, larva, pupa, and adult) (Fig. 4 and Fig. S4).

**Fig. 5:**
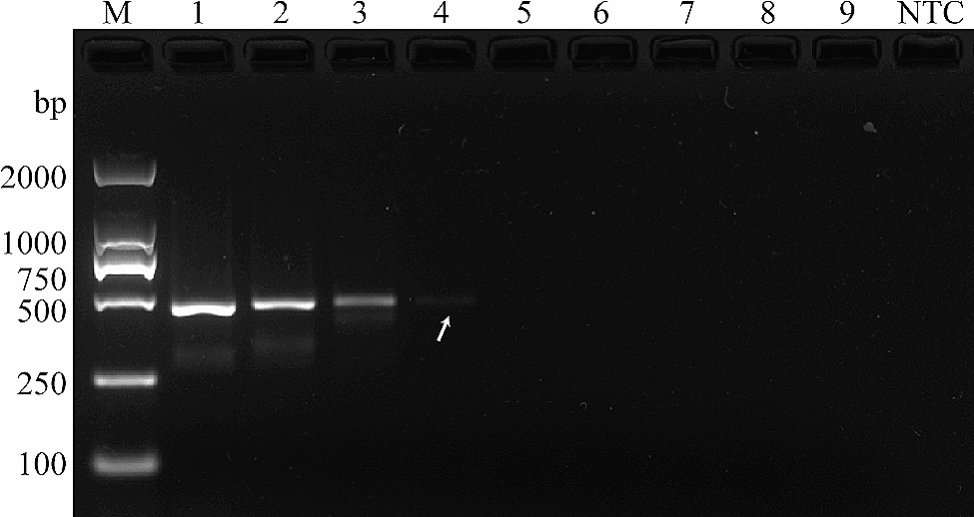
Sensitivity of the primers targeting the *COI* gene. 1–9: 46.2 ng/µL, 4.62 ng/µL, 0.46 ng/µL, 46 pg/µL, 4.6 pg/µL, 0.46 pg/µL, 46 fg/µL, 4.6 fg/µL, and 0.46 fg/µL; NTC: no template control

We assessed the sensitivity of the four primers using diluted *H. ligniperda* DNA templates. The results indicate that the detection thresholds for the four primers are as follows: 46 pg/µL (*COI*), 0.46 ng/µL (*ND2*), 46 pg/µL (*ND4*), and 0.46 ng/µL (*ND5*) (Fig. 5 and Fig. S5). The experimental results demonstrate that all four primers exhibit excellent specificity, broad stability, and satisfactory performance. In addition, no significant differences in specificity and sensitivity were found among target genes at different evolutionary rates.

### Application of detection of trapped samples

**Fig. 6:**
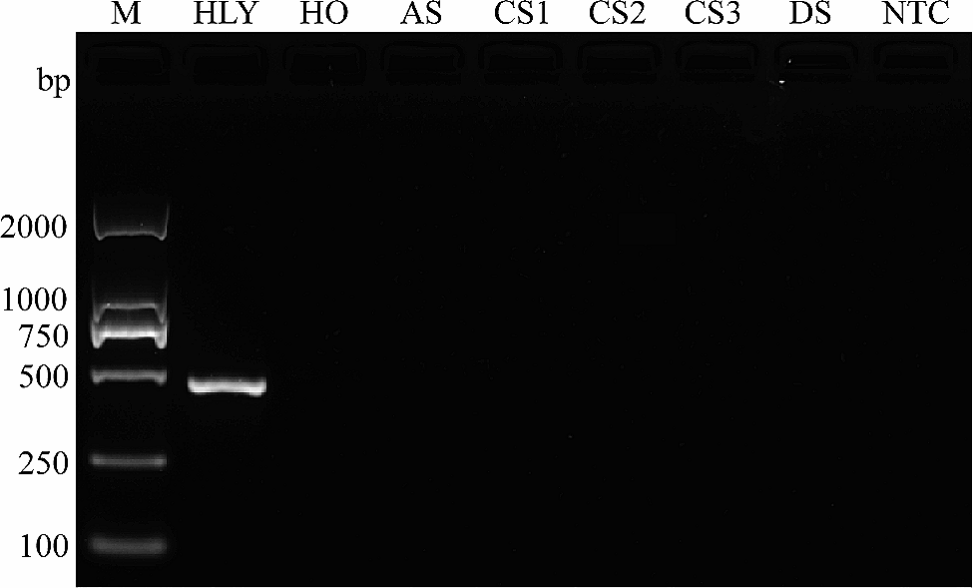
Testing the primers targeting the *COI* gene in a wider range of species. M: DL2000 DNA marker; HLY: *H. ligniperda*, Yantai; HO: *Hylastes opacus*; AS: *Ambrosiophilus* sp.; CS1: *Cardiophorus* sp.; CS2: *Cardiophorus* sp.; CS3: *Cyrtogenius* sp.; DS: *Dendroctonus* sp.; NTC: no template control

We continuously monitored beetles with traps in the same area in April and May 2022. Based on morphological characteristics, we classified the bark beetles obtained by trapping into seven species. Seven species were identified using SS-PCR with four primers, and only one band was displayed in the electrophoresis results (Fig. 6 and Fig. S6). We used DNA barcoding to further identify these species. The identified species included *H. ligniperda*, *H. opacus*, *Ambrosiophilus* sp., *Cardiophorus* sp., *Cardiophorus* sp., *Cyrtogenius* sp., and *Dendroctonus* sp., respectively. Consequently, the results indicate that the SS-PCR method based on four primers are advantageous and perform well in practical applications. Moreover, target genes at different evolutionary rates show consistent results.

## Discussion

Biological invasions are a form of global change threatening biodiversity, ecosystem stability, and human health [38]. The accelerating pace of commercial and social globalization creates unprecedented opportunities to migrate bark beetles to new areas. Once established in previously unoccupied areas, bark beetles can be nearly impossible to eradicate [39]. The complex distribution and fauna of bark beetle species in China and the invasions of alien bark beetle species threated the development of forestry [40]. An effective and affordable detection method is critical for incipient populations of invasive species. The identification of key pests is the basis for further scientific management. This technology will greatly improve the efficiency of pest control.

The *H. ligniperda* beetle has recently been found to have successfully invaded China, and its adults have a strong ability to diffuse. These beetles spread to all planting areas of *Pinus radiata* within five years after their invasion in Chile [13], and New Zealand has only three years since the first discovery of *H. ligniperda* spread throughout the country [41]. Furthermore, *H. ligniperda* adapts quickly to the new environment and tends to become a regional advantage in newly introduced areas [42–44]. Identification of alien invasive species is a necessary prerequisite for effectively preventing them. Therefore, early monitoring of the *H. ligniperda* population in China should be given sufficient attention.

The rapid advancement of molecular biology technologies has led to the development of species identification-related techniques, with abundant and accurate nucleotide data making molecular identification of target species more efficient. Currently, *H. ligniperda* has less nucleic acid sequence information, including species identification [22], molecular diagnosis by reversed dot blot method (PCR-RDB) [45], construction of DNA barcode banks [46], and phylogenetic analysis [34, 35]. These sequences are helpful for species identification and detection. Mitochondrial sequences have been extensively employed as target genes for molecular species identification, enhancing the precision and probability of pest detection [47]. We sequenced and annotated the mitochondrial genome of *H. ligniperda* for screening molecular detection targets and enriched the nucleotide dataset. The phylogenetic relationship between 44SPE and the genetic distances result among 9SPE indicated that *T. piniperda*, *D. valens*, and *D. micans* are more closely related to *H. ligniperda*. Furthermore, these species are remarkably similar in morphology, we tested the specificity of the developed SS-PCR method with there species in preliminary trials.

The stability of a molecular detection system is primarily contingent on the stability of the target gene. Mitochondrial genes are particularly advantageous for enhancing resolution at lower classification levels [33]. Regions that exhibit conservation within species while also displaying substantial interspecies nucleotide variation are frequently employed to develop species-specific markers, and this is especially applicable in situations involving closely related species that share significant morphological similarities [24]. Generally, NADH genes show greater nucleotide substitution rates and more variation than cytochrome oxidase genes [48]. The *COI* gene is a frequently used universal barcode fragment for species classification [49].Some research data suggest that genes with rapid evolutionary rates might be more suitable targets for interspecies identification [32, 33]. However, the *COI* gene displayed the least variability and the slowest evolutionary rates among all the genes examined in our current dataset. Additionally, we selected three genes (e.g. *ND2*, *ND4*, and *ND5*) known for their fast evolutionary rates and fragment lengths exceeding 1,000 bp as targets for our SS-PCR assay. This choice aimed to investigate whether the detection performance of the method correlated with the evolutionary rates of the selected genes. Our findings indicate that the evolutionary rate of genes used in the SS-PCR method may not necessarily be linked to the design of specific primers. Subsequent comprehensive studies have confirmed these results, affirming the specificity, sensitivity, and overall effectiveness of the four primers in suitability tests.

Despite the development and utilization of more advanced identification methods, such as loop-mediated isothermal amplification (LAMP) and recombinase polymerase amplification (RPA), SS-PCR has been widely utilized for the rapid detection of pest species [4, 24, 26–29]. However, these methods also have their own set of shortcomings. For instance, LAMP relies on fluorescent dyes or metal indicators, which might be influenced by subjective factors related to individual vision [50]. On the other hand, although the RPA assay results can be interpreted using lateral flow dipsticks, they necessitate the purchase of expensive commercial kits, making experiments cost-prohibitive [51]. In addition, their high sensitivity and amplification capabilities may increase the risk of false positives and difficulties in interpretation of results [52]. In contrast, the SS-PCR method offers a cost-effective solution while maintaining high performance. Furthermore, it is relatively resistant to aerosol contamination, addressing a common issue observed in many isothermal amplification techniques [53]. In another study, we found that the selection of target genes in higher-resolution detection technologies may be related to their evolutionary, as these technologies are more sensitive to detecting individual base pair matching sites, such as RPA [53].

## Conclusions

**Fig. 7:**
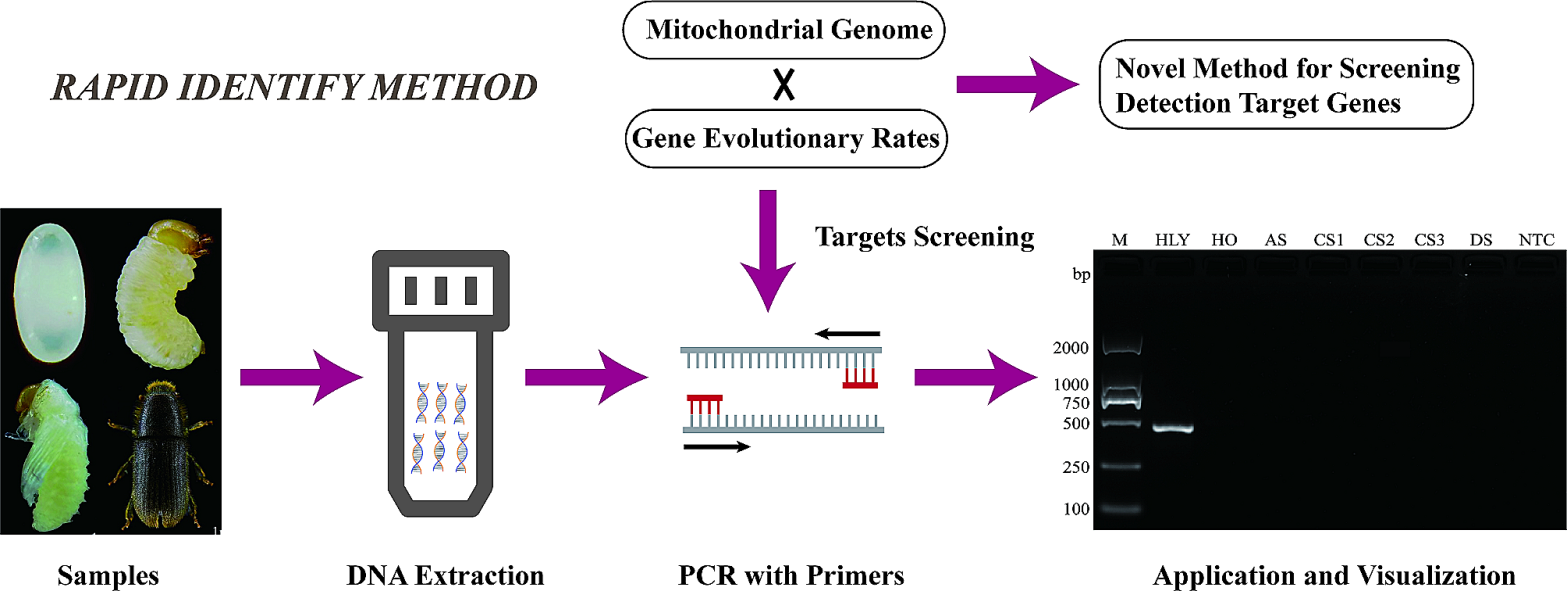
Development of the SS-PCR method based on mitochondrial genome

In summary, the SS-PCR was developed based on four single mitochondrial genes has been proven effective for identifying *H. ligniperda*, successfully addressing variations in different sexes, developmental stages, or phenotypes (Fig. 7). This method is simple, straightforward to interpret, and cost-effective. The entire process can be performed within 2–3 h. Moreover, we proposed a novel method for screening targets for rapid detection using the evolutionary rate of the gene. Our method contributes to the ongoing monitoring and management of large-scale analyses of early *H. ligniperda* populations, aiding in the surveillance of *H. ligniperda* that may appear in imported and exported pinewood and woody materials. This undoubtedly enhances the security of international trade, holds significant importance in restraining the global spread of *H. ligniperda*, ensures the sustainable management of pine tree resources.

## Methods

### Samples and DNA extraction

Individuals of *H. ligniperda* were collected in four cities in China (WH, YT, TA, and QD) (Table 1). Pheromone traps attracted *H. ligniperda* adults in each pine forest, while other developmental stages were obtained from under the bark of the pines. Species identification by morphology and DNA barcoding first. All samples were collected in centrifugal tubes containing anhydrous ethanol and stored at − 20℃. Genomic DNA was extracted from the thorax and leg muscle tissues using the TIANamp Genomic DNA Kit (TIANGEN, China) according to the manufacturer’s protocol. The extracted DNA was stored at − 20℃ for future use.

**Table 1:** Samples information in the present study

Code^*^	Species	Developmental stage	Locality	Date
HLY	*Hylurgus ligniperda*	Adult	Yantai, Shandong	July, 2022
HLW	*Hylurgus ligniperda*	Adult	Weihai, Shandong	April, 2022
HLQ	*Hylurgus ligniperda*	Adult	Qingdao, Shandong	October, 2022
HLT	*Hylurgus ligniperda*	Adult	Tai’an, Shandong	May, 2022
HLE	*Hylurgus ligniperda*	Egg	Yantai, Shandong	May, 2023
HLL	*Hylurgus ligniperda*	Larvae	Yantai, Shandong	July, 2022
HLP	*Hylurgus ligniperda*	Pupae	Yantai, Shandong	July, 2022
TP	*Tomicus piniperda*	Adult	Weihai, Shandong	April, 2022
DV	*Dendroctonus valens*	Adult	Beijing	March, 2023
DM	*Dendroctonus micans*	Adult	Maixiu, Qinghai	August, 2021
HO	*Hylastes opacus*	Adult	Weihai, Shandong	April, 2022
AS	*Ambrosiophilus* sp.	Adult	Weihai, Shandong	May, 2022
CS1	*Cardiophorus* sp.	Adult	Weihai, Shandong	April, 2022
CS2	*Cardiophorus* sp.	Adult	Weihai, Shandong	April, 2022
CS3	*Cyrtogenius* sp.	Adult	Weihai, Shandong	April, 2022
DS	*Dendroctonus* sp.	Adult	Weihai, Shandong	May, 2022

^*^The code was used in the following figure legends

### Mitochondrial genome sequencing, assembly, and genome annotation

Next-generation sequencing libraries were constructed with an average insert size of 350 bp and sequenced on an Illumina NovaSeq 6000 platform (San Diego, California, USA) with 150 bp paired-end reads at Annaroad Gene Technology (Beijing) Co., Ltd. Clean reads were obtained after trimming adapters using Trimmomatic and filtering low-quality or short reads using Prinseq. The genome was *de nova* assembled using Geneious Prime 2020 (https://www.geneious.com/prime) and IDBA-UD assembler software [32, 47]. Protein-coding genes (PCGs), rRNA, and tRNA genes were annotated using the MITOS WebServer with the following parameters: Reference = “RefSeq 63 Metazoa” and Genetic Code = “5 Invertebrate”. The boundaries of the PCGs and rRNA genes were determined by alignment with the positions of homologous genes and tRNA genes reported in other Curculionidae families. The start and stop codons and the length of each PCG were manually confirmed and modified. A circular map of the complete mitochondrial genome map was made by Poskee [54]. The nucleotide composition of the *H. ligniperda* mitochondrial genome was determined using MEGA X [55]. The base composition skew was analyzed by manual calculation, and the calculations were performed according to the following formula: AT-skew = [A - T]/[A + T] and GC-skew = [G-C]/[G + C] [56].

### Phylogenetic analysis and rate of evolution analysis

The following analysis used three datasets: (i) 44 species of bark beetles (44SPE), (ii) nine species in Hylesininae (9SPE), and (iii) *H. ligniperda*, *T. piniperda*, *D. micans*, and *D. valens* (4SPE). The taxonomic system used in this study is referenced in previous works [40, 57].

The phylogenetic tree was constructed based on 13 PCGs of 44SPE, with *Curculio davidi* and *C. Davidi* serving as outgroups. All complete mitochondrial genomes of 44SPE were downloaded from the NCBI (Table S1). Some of these species of mitochondrial genome were re-annotated. Phylogenetic analysis of taxa within bark beetles was conducted in PhyloSuite v1.2.2 [33, 58]. The 13 PCGs of 44SPE were aligned using the MAFFT algorithm individually. The sequences were trimmed in trimAL v1.2. The 13 PCGs were concatenated using FASconCAT-G v. 1.04. Finally, a dataset with 11,021 nucleotides was conducted. Maximum likelihood (ML) phylogenies were inferred using IQ-TREE under the partition schemes, and best-fit models (edge-linked) were determined by ModelFinder using the corrected Akaike information criterion. The node support was evaluated using 1000 ultrafast bootstrap replicates. Bayesian inference (BI) phylogenies were inferred using MrBayes with two independent runs, each with four Markov chain Monte Carlo chains (one cold) for 5,000,000 generations, with sampling every 1000 generations. The first 25% of the runs were discarded as burn-in before estimating posterior probabilities for branch support. The best-fit partitioning schemes (edge-linked) and substitution models were determined using ModelFinder and the corrected Bayesian information criterion.

We calculated the genetic distances among 9SPE based on 13 PCGs using MEGA X with the Kinmura-2-parameter model [55]. Nucleotide diversity (Pi) values of each PCG from 4SPE were determined using sliding window analyses with a sliding window of 200 bp and a step size of 20 bp in DnaSP v6 [59]. The average genetic distances of each PCG from 4SPE under the Kinmura-2-parameter model were calculated using MEGA X [55]. The rate of evolution of each PCG (Ka/Ks) from 4SPE was calculated using DnaSP v6 [59].

### Species-specific primer design

The target sequences of 4SPE were aligned and manually edited using ClustalW in MEGA X [55]. Primer Premier 5.0 was utilized for the primer design process [60]. The primers were designed based on regions of the target sequences (*COI*, *ND2*, *ND4*, and *ND5*) that exhibited conservation within *H. ligniperda* while showing substantial differences from other species (Table 2). The primers were synthesized by Beijing Tsingke Biotech Co., Ltd. (Beijing, China).

**Table 2:** Primer sequences used in the species-specific PCR assay

Gene	Primers	Sequences (5’-3’)	Length (bp)	Production (bp)	Annealing (°C)
DNA barcoding	LCO1490	GGTCAACAAATCATAAAGATATTGG	25	/	43
	HCO2198	TAAACTTCAGGGTGACCAAAAAATCA	26		
*COI*	HLCOIF	CTTCTTTAACCTTTCTCT	18	474	55
	HLCOIR	TTTTTCCTCTCTCTTGTC	18		
*ND2*	HLND2F	TTCTGGCTCCCTGAAGTA	18	434	55
	HLND2R	CCTAAAAATGGGGGTAAA	18		
*ND4*	HLND4F	TTTTAGGATGAGGTAGACA	19	346	56
	HLND4R	ATACCAAAATCAATAAACAA	20		
*ND5*	HLND5F	TGCCCTATCTAACCGTGT	18	452	56
	HLND5R	ATTCTGCCCCACCTACAC	18		

### Specificity and sensitivity of the SS-PCR method

The PCR reactions were conducted in a total reaction volume of 25 µL, which included 1.5 µL of DNA, 1 µL each of forward and reverse primers (10 µmol/L), 12.5 µL of 2× Flash PCR MasterMix (CWBIO), and 9 µL of ddH_2_O. The thermal cycling program for the reactions was as follows: initial denaturation at 98℃ for 1 min, followed by 34 cycles of denaturation at 94℃ for 10 s, extension at 72℃ for 30 s, and a final elongation at 72℃ for 1 min (Table 2). The DNA was replaced with ddH_2_O as the no template control (NTC). The 2 µL of PCR product was visualized with 1.5% agarose gel electrophoresis at 125 V for 25 min with 1 × TAE as the electrophoresis buffer.

The specificity of the primers was evaluated among 4SPE, and the DNA from 4SPE was prescreened for usability using universal primers LCO1490 and HCO2198. The broad applicability and specificity of the primers were assessed using *H. ligniperda* samples from various developmental stages and diverse geographical populations. Diluted DNA (HLY) was used to determine the sensitivity of the primers. The 10-fold dilutions of the DNA used were as follows: 46.2 ng/µL, 4.62 ng/µL, 0.46 ng/µL, 46 pg/µL, 4.6 pg/µL, 0.46 pg/µL, 46 fg/µL, 4.6 fg/µL, and 0.46 fg/µL.

### The SS‒PCR method was applied to a wide range of species

During the investigation of *H. ligniperda*, several other bark beetles were also trapped. These beetles were morphologically classified into seven species using a microscope. The identification results were confirmed using agarose gel electrophoresis. Finally, the reliability of the SS‒PCR method was validated by DNA barcoding.

### Electronic supplementary material

Below is the link to the electronic supplementary material.

**Supplementary Material: Table S1.** The species included in the phylogenetic analysis. **Table S2.** Structure of the mitochondrial genome of Hylurgus ligniperda. *Negative numbers indicate that adjacent genes overlap. **Table S3.** Base composition in the mitochondrial genome of *Hylurgus ligniperda*. **Figure S1.** The phylogenetic tree of *Hylurgus ligniperda* inferred from 44SPE using Bayesian inference. **Figure S2.** Genetic distance heat map of Hylesininae among 9SPE. **Figure S3.** PCR products of DNA barcoding of four species. **Figure S4.** Specificity and stability of the primers targeting the *ND2*, *ND4*, and *ND5* genes. M: DL2000 DNA marker; HLY: *H. ligniperda*, Yantai; HLW: *H. ligniperda*, Weihai; HLQ: *H. ligniperda*, Qingdao; HLT: *H. ligniperda*, Tai’an; HLE: *H. ligniperda*, egg; HLL: *H. ligniperda*, larvae; HLP: *H. ligniperda*, pupae; TP: *T. piniperda*; DV: *D. valens*; DM: *D. micans*; NTC: no template control. **Figure S5.** Sensitivity of the primers targeting the *ND2*, *ND4*, and *ND5* genes. 1–9: 46.2 ng/µL, 4.62 ng/µL, 0.46 ng/µL, 46 pg/µL, 4.6 pg/µL, 0.46 pg/µL, 46 fg/µL, 4.6 fg/µL, and 0.46 fg/µL; NTC: no template control. **Figure S6.** Testing in a wider range of species using primers targeting *ND2*, *ND4*, and *ND5* genes. M: DL2000 DNA marker; HLY: *H. ligniperda*, Yantai; HO: *Hylastes opacus*; AS: *Ambrosiophilus* sp.; CS1: *Cardiophorus* sp.; CS2: *Cardiophorus* sp.; CS3: *Cyrtogenius* sp.; DS: *Dendroctonus* sp.; NTC: no template control

## Data Availability

The mitochondrial genome sequence have been deposited in NCBI (https://www.ncbi.nlm.nih.gov/) with accession number: OR105874.
