# Supplementary material for: Mitochondrial genome provides species-specific targets for the rapid detection of early invasive populations of Hylurgus ligniperda in China
Source: BMC Genomics. 2024 Jan 22;25:90. doi: 10.1186/s12864-024-10011-z (PMC10804472; doi:10.1186/s12864-024-10011-z)
Supplement: Supplementary file 1 — Supplementary Material: Table S1. The species included in the phylogenetic analysis. Table S2. Structure of the mitochondrial genome of Hylurgus ligniperda. *Negative numbers indicate that adjacent genes overlap. Table S3. Base composition in the mitochondrial genome of Hylurgus ligniperda. Figure S1. The phylogenetic tree of Hylurgus ligniperda inferred from 44SPE using Bayesian inference. Figure S2. Genetic distance heat map of Hylesininae among 9SPE. Figure S3. PCR products of DNA barcoding of four species. Figure S4. Specificity and stability of the primers targeting the ND2, ND4, and ND5 genes. M: DL2000 DNA marker; HLY: H. ligniperda, Yantai; HLW: H. ligniperda, Weihai; HLQ: H. ligniperda, Qingdao; HLT: H. ligniperda, Tai’an; HLE: H. ligniperda, egg; HLL: H. ligniperda, larvae; HLP: H. ligniperda, pupae; TP: T. piniperda; DV: D. valens; DM: D. micans; NTC: no template control. Figure S5. Sensitivity of the primers targeting the ND2, ND4, and ND5 genes. 1–9: 46.2 ng/µL, 4.62 ng/µL, 0.46 ng/µL, 46 pg/µL, 4.6 pg/µL, 0.46 pg/µL, 46 fg/µL, 4.6 fg/µL, and 0.46 fg/µL; NTC: no template control. Figure S6. Testing in a wider range of species using primers targeting ND2, ND4, and ND5 genes. M: DL2000 DNA marker; HLY: H. ligniperda, Yantai; HO: Hylastes opacus; AS: Ambrosiophilus sp.; CS1: Cardiophorus sp.; CS2: Cardiophorus sp.; CS3: Cyrtogenius sp.; DS: Dendroctonus sp.; NTC: no template control [file 12864_2024_10011_MOESM1_ESM.pdf]

Table S1 The species included in the phylogenetic analysis.

Family	Subfamily	Genus	Species	Accession Number
In Groups				
Curculionidae	Scolytinae	*Scolytus*	*Scolytus seulensis*	NC_046588
			Scolytus schevyrewi	NC_046589
	Hylesininae	*Polygraphus*	*Polygraphus poligraphus*	NC_060713
		Tomicus	*Tomicus piniperda*	MH281570
		Dendroctonus	*Dendroctonus micans*	MZ768861
			Dendroctonus valens	NC_061915
		Hylurgus	*Hylurgus ligniperda*	OR105874
		Hylastes	*Hylastes attenuatus*	NC_036290
			Hylastes brunneus	NC_036262
			Hylastes opacus	KX035200
		Phloeosinus	*Phloeosinus perlatus*	NC_057470
	Scolytoplatypodinae	*Scolytoplatypus*	*Scolytoplatypus wugongshanensis*	OP712675
			Scolytoplatypus skyliuae	OP719283
			Scolytoplatypus sinensis	OP719284
			Scolytoplatypus raja	OP719285
	Ipinae	*Hypothenemus*	*Hypothenemus* sp. KM-2015	KX035224
			Hypothenemus sp. BMNH 1040235	KX035186
			Hypothenemus sp. BMNH 1040003	KX035175
			Hypothenemus sp. BMNH 1039866	KX035165
			Hypothenemus sp. BMNH 1039837	KX035163
		Trypodendron	*Trypodendron domesticum*	NC_036286
			Trypodendron signatum	NC_036292
		Coptodryas	*Coptodryas elegans*	OK539700
		Euwallacea	*Euwallacea fornicatus*	NC_059702
			Euwallacea interjectus	NC_069051
		Xyleborus	*Xyleborus* sp. BMNH 1040067	KX035179
		Xylosandrus	*Xylosandrus crassiusculus*	NC_036284
			Xylosandrus germanus	NC_036280
			Xylosandrus morigerus	NC_036283
			Xylosandrus sp.	ON169991
		Dryocoetes	*Dryocoetes autographus*	NC_036287
			Dryocoetes hectographus	NC_062125
			Dryocoetes villosus	NC_036282
		Pityophthorus	*Pityophthorus pubescens*	KX035209
		Pityogenes	*Pityogenes bidentatus*	KX035211
		Orthotomicus	*Orthotomicus erosus*	MZ823388
			Orthotomicus laricis	NC_036291
		Ips	*Ips acuminatus*	MK988441
			Ips calligraphus	NC_060365
			Ips hauseri	MZ768860
			Ips nitidus	NC_062131

			Ips sexdentatus	KX035215
			Ips subelongatus	MZ766130
			Ips typographus	MZ766131
Out Groups				
Curculionidae	Curculioninae	*Curculio*	*Curculio davidi*	NC_034293
			Curculio elephas	KX087269

Table S2 Structure of the mitochondrial genome of *Hylurgus ligniperda*.

Gene	Direction	Location	Length (bp)	Anticodon	Start codon	Stop codon	Intergenic Nucleotides*
ND5	F	1-1,714	1,714		ATT	T-	
*tRNA^Phe^*	F	1,712-1,775	64	GAA			-3
*tRNA^Glu^*	R	1,776-1,841	66	TTC			0
*tRNA^Ser(AGN)^*	R	1,847-1,912	66	TCT			5
*tRNA^Asn^*	R	1,913-1,977	65	GTT			0
*tRNA^Arg^*	R	1,976-2,042	67	TCG			-2
*tRNA^Ala^*	R	2,042-2,104	63	TGC			-1
ND3	R	2,104-2,457	354		ATA	TAA	-1
*tRNA^Gly^*	R	2,458-2,523	66	TCC			0
COIII	R	2,527-3,309	783		ATG	TAA	3
ATP6	R	3,309-3,986	678		ATG	TAA	-1
ATP8	R	3,980-4,135	156		ATT	TAA	-7
*tRNA^Asp^*	R	4,136-4,198	63	GTC			0
*tRNA^Lys^*	R	4,198-4,265	68	CTT			-1
COII	R	4,270-4,956	687		ATT	TAA	4
*tRNA^Leu(UUR)^*	R	4,957-5,021	65	TAA			0
COI	R	5,017-6,585	1,569		ATT	TAA	-5
*tRNA^Tyr^*	F	6,557-6,621	65	GTA			-29
*tRNA^Cys^*	F	6,627-6,690	64	GCA			5
*tRNA^Trp^*	R	6,690-6,754	65	TCA			-1
ND2	R	6,753-7,760	1,008		ATT	TAA	-2
*tRNA^Met^*	R	7,766-7,833	68	CAT			5
*tRNA^Gln^*	F	7,840-7,908	69	TTG			6
*tRNA^Ile^*	R	8,624-8,689	66	GAT			0
Control region	F	8,690-10,510	1,821				715
srRNA	F	10,511-11,269	759				0
*tRNA^Val^*	F	11,268-11,333	66	TAC			-2
lrRNA	F	11,353-12,652	1,300				19
*tRNA^Leu(CUN)^*	F	12,630-12,693	64	TAG			-23
ND1	F	12,713-13,642	930		ATA	TAA	19
*tRNA^Ser(UCN)^*	R	13,660-13,728	69	TGA			17
CYTB	R	13,728-14,867	1,140		ATG	TAA	-1
ND6	R	14,867-15,373	507		ATT	TAA	-1
*tRNA^Pro^*	F	15,376-15,441	66	TGG			2
*tRNA^Thr^*	R	15,442-15,506	65	TGT			0
ND4L	F	15,515-15,808	294		ATG	TAG	8
ND4	F	15,812-17,155	1,344		ATG	TAA	3
*tRNA^His^*	F	17,139-17,202	64	GTG			-17

* Negative numbers indicate that adjacent genes overlap.

Table S3 Base composition in the mitochondrial genome of *Hylurgus ligniperda*.

**Gene**	**Length (bp)**	**A %**	**T %**	**AT %**	**AT-skew**	**G %**	**C %**	**GC %**	**GC-skew**
All gene	17,202	36.3	40.6	76.9	-0.0560	14.4	8.8	23.2	0.2414
13 PCGs	11,164	31.9	42.6	74.5	-0.1436	11.9	13.7	25.5	-0.0706
rRNA	2,059	37.7	42.4	80.1	-0.0587	13.6	6.3	19.9	0.3668
tRNA	1,444	40.6	38.6	79.2	0.02525	11.4	9.4	20.8	0.0962
Control region	1,821	39.0	44.6	83.6	-0.0670	8.4	8.0	16.4	0.0244

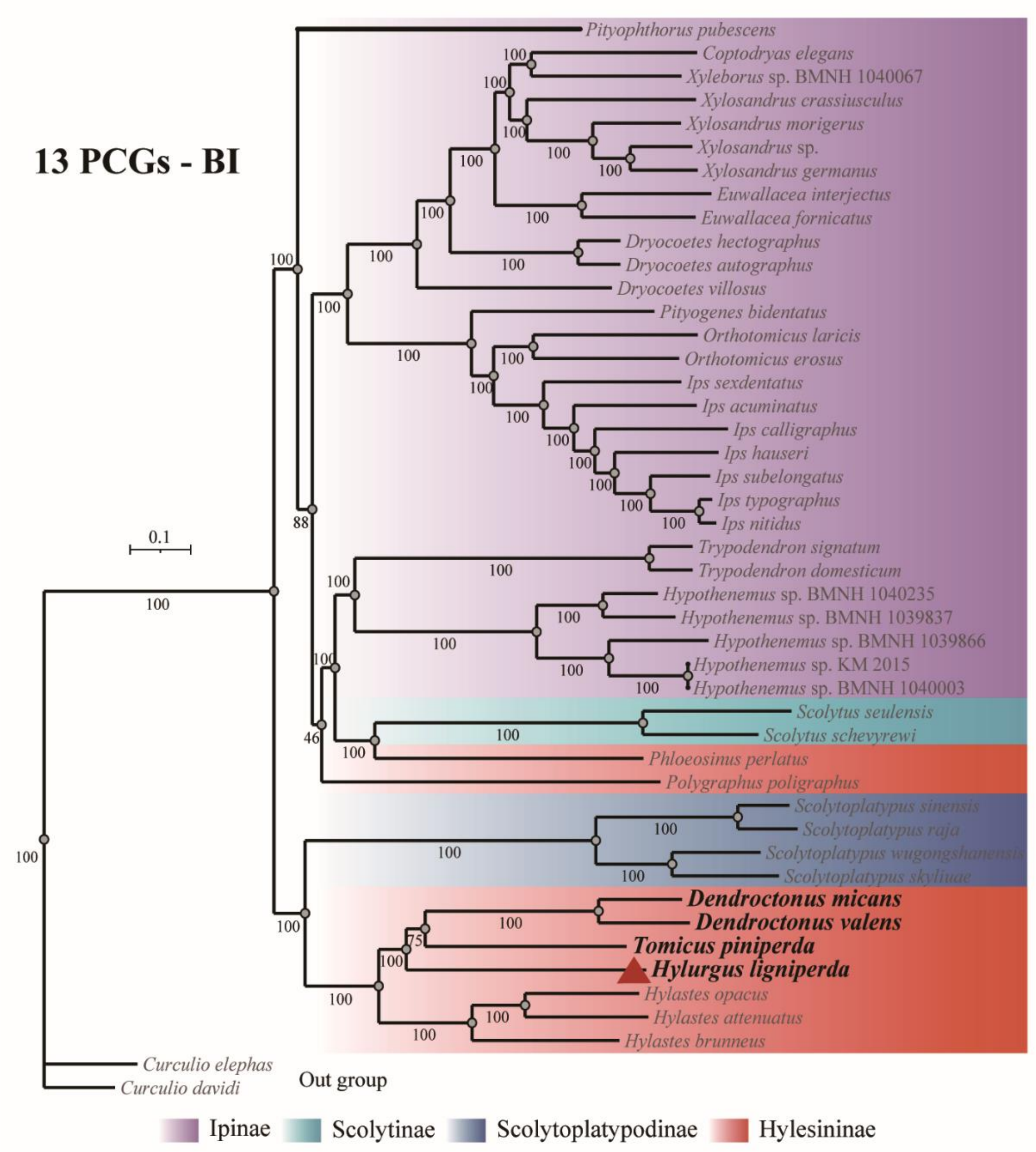

Figure S1 The phylogenetic tree of *Hylurgus ligniperda* inferred from 44SPE using Bayesian inference.

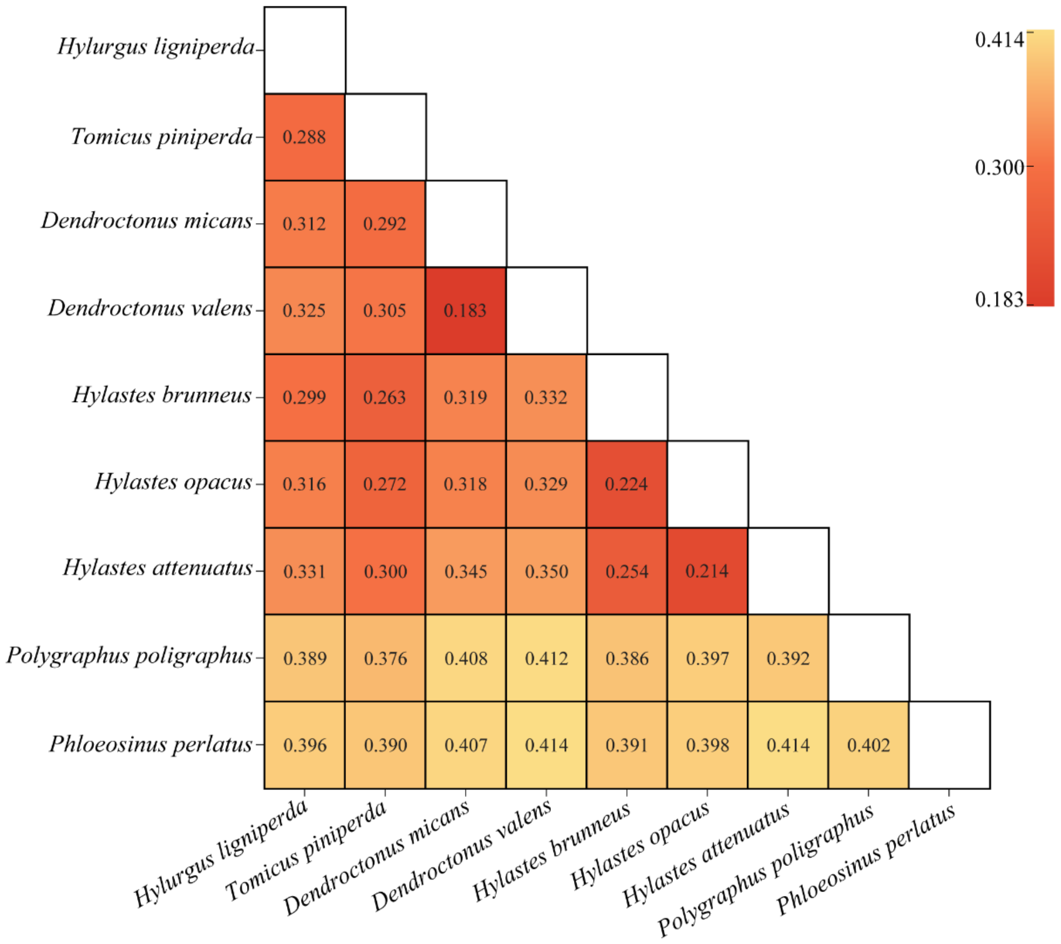

Figure S2 Genetic distance heat map of Hylesininae among 9SPE.

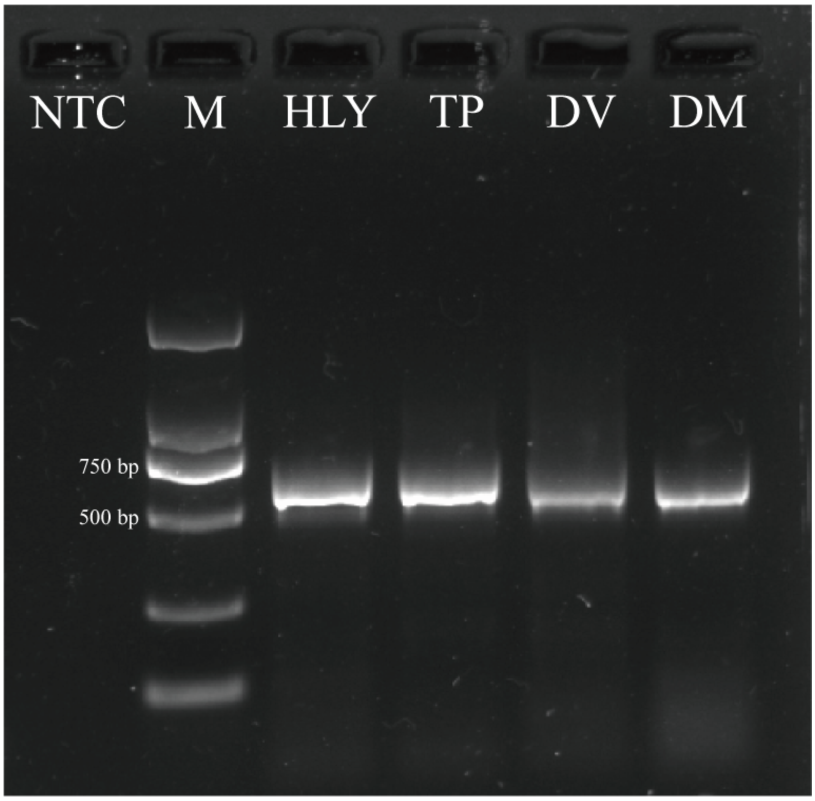

Figure S3 PCR products of DNA barcoding of four species.

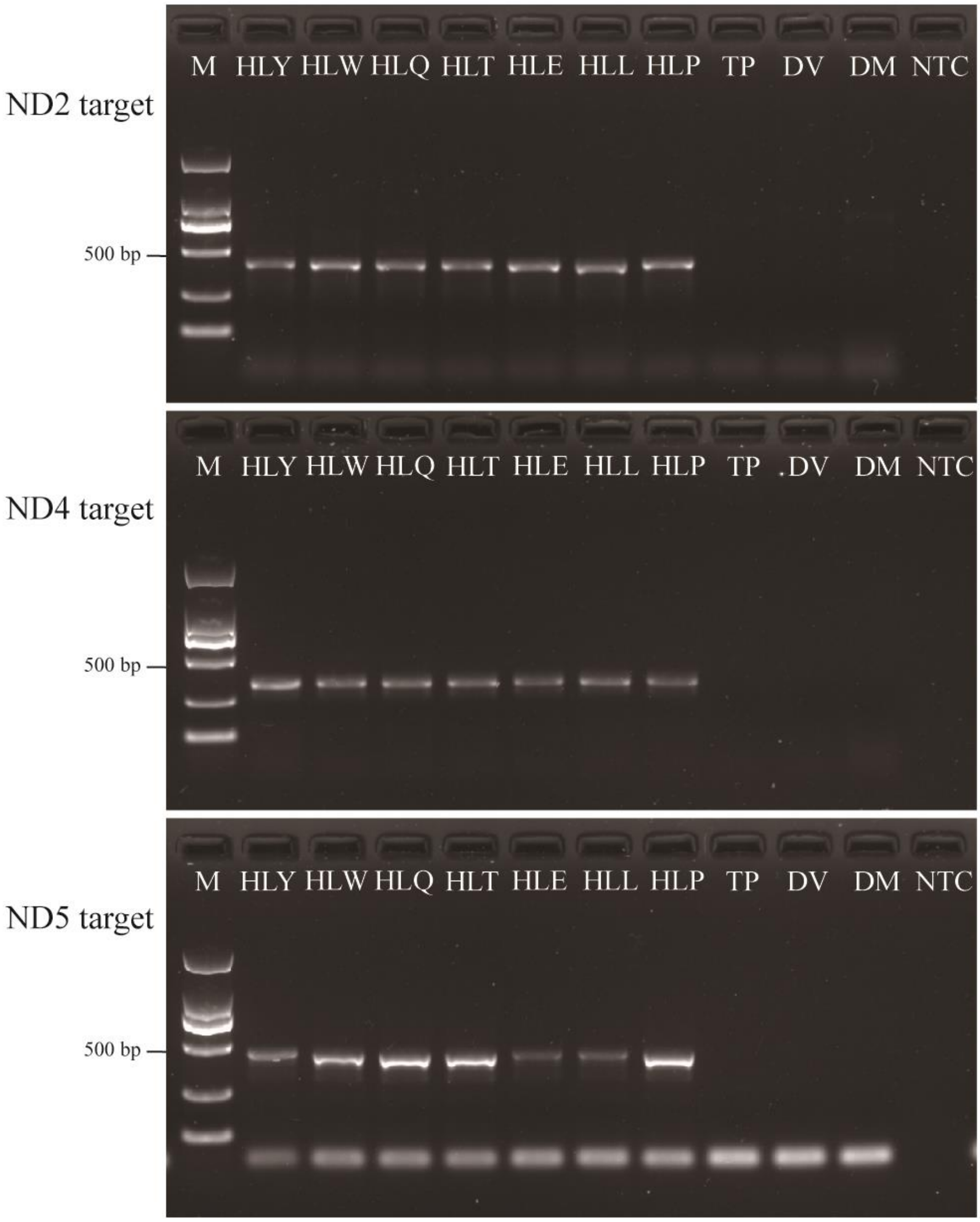

Figure S4 Specificity and stability of the primers targeting the *ND2*, *ND4*, and *ND5* genes. M: DL2000 DNA marker; HLY: *H. ligniperda*, Yantai; HLW: *H. ligniperda*, Weihai; HLQ: *H. ligniperda*, Qingdao; HLT: *H. ligniperda*, Tai'an; HLE: *H. ligniperda*, egg; HLL: *H. ligniperda*, larvae; HLP: *H. ligniperda*, pupae; TP: *T. piniperda*; DV: *D. valens*; DM: *D. micans*; NTC: no template control.

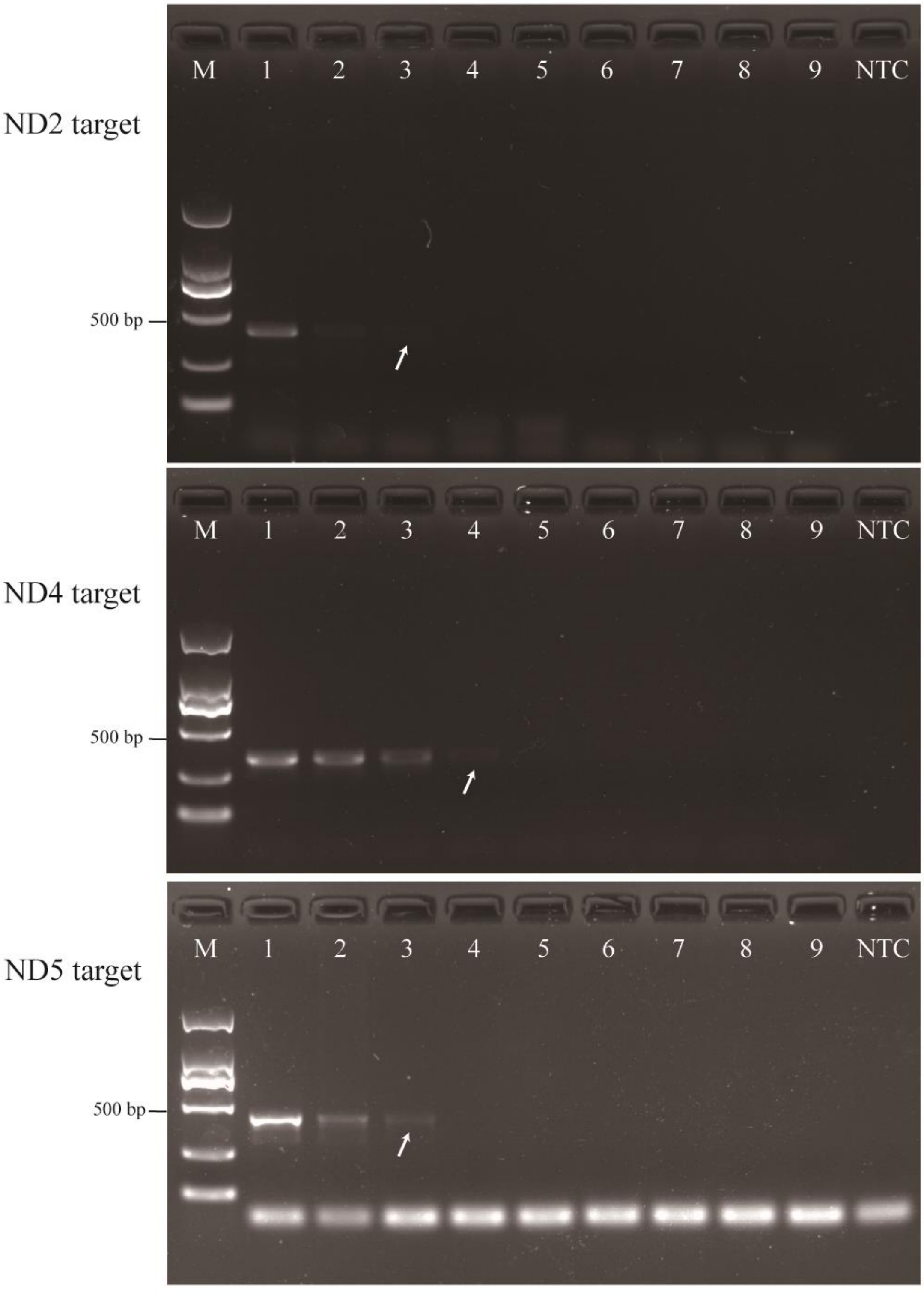

Figure S5 Sensitivity of the primers targeting the *ND2*, *ND4*, and *ND5* genes. 1-9: 46.2 ng/μL, 4.62 ng/μL, 0.46 ng/μL, 46 pg/μL, 4.6 pg/μL, 0.46 pg/μL, 46 fg/μL, 4.6 fg/μL, and 0.46 fg/μL; NTC: no template control.

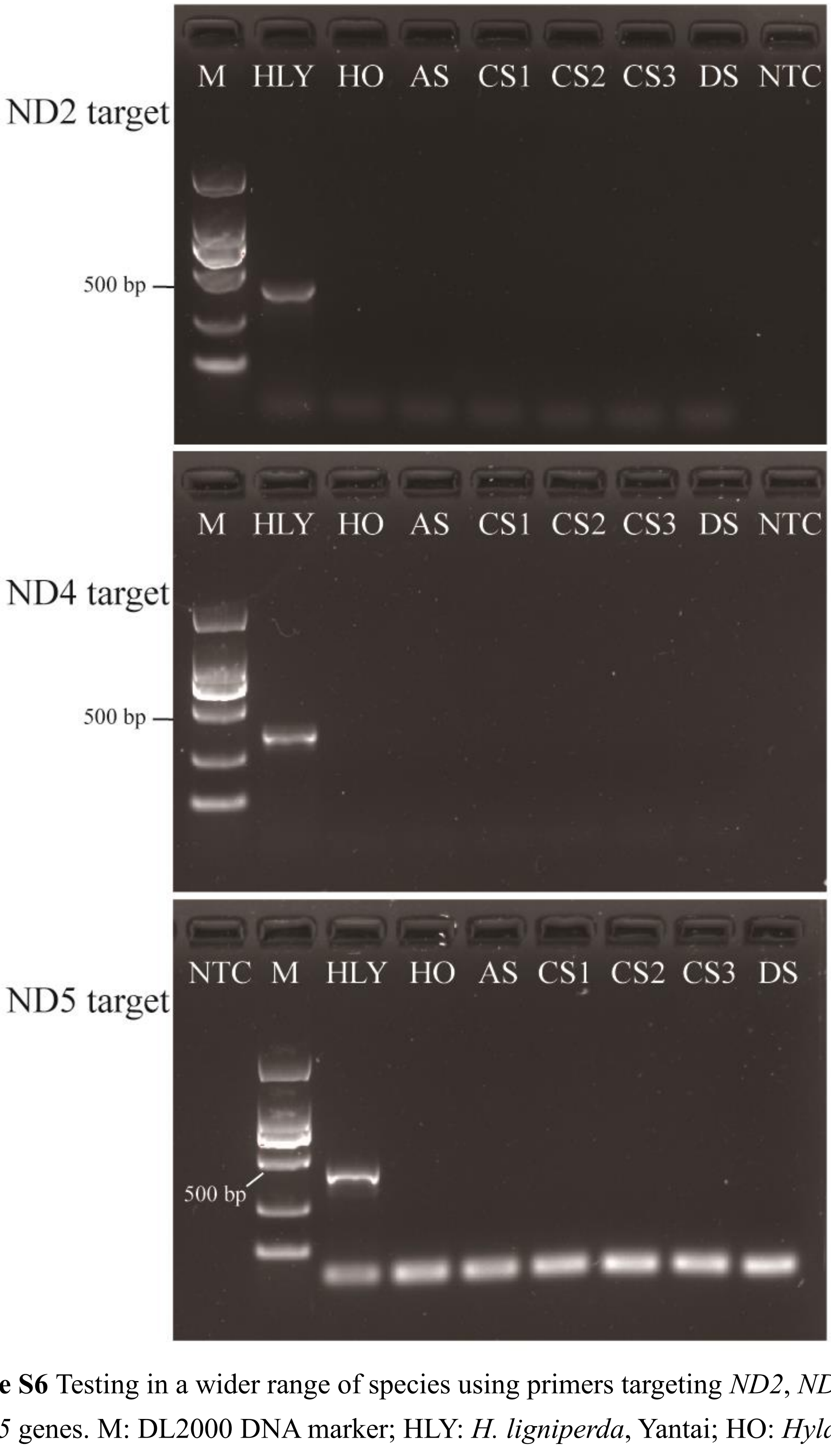

Figure S6 Testing in a wider range of species using primers targeting *ND2*, *ND4*, and *ND5* genes. M: DL2000 DNA marker; HLY: *H. ligniperda*, Yantai; HO: *Hylastes opacus*; AS: *Ambrosiophilus* sp.; CS1: *Cardiophorus* sp.; CS2: *Cardiophorus* sp.; CS3: *Cyrtogenius* sp.; DS: *Dendroctonus* sp.; NTC: no template control.
